# Supplementary material for: Lipoic acid rejuvenates aged intestinal stem cells by preventing age‐associated endosome reduction
Source: EMBO Rep. 2020 Jul 9;21(8):e49583. doi: 10.15252/embr.201949583 (PMC7403706; doi:10.15252/embr.201949583)
Supplement: Supplementary file 1 — Expanded View Figures PDF [file EMBR-21-e49583-s001.pdf]

Expanded View Figures

Figure EV1. Alpha-lipoic acid (ALA) synthesis reduces in aged *Drosophila* midguts, and orally administered ALA rejuvenates aged intestinal stem cells (ISCs; related to Fig 1).

A Model of *Drosophila* intestinal stem cell (ISC) lineages. One ISC (Dl^+ and Esg^+) produces a new ISC and differentiates into a diploid precursor enteroblast (EB; Esg^+ and Su(H)GBE^+) with high Notch or a diploid precursor enteroendocrine mother cell (EMC). The EMC divides once to produce a pair of diploid enteroendocrine cells (EEs; $Pros^+$). The post-mitotic EB further differentiates into pre-enterocyte (pre-EC; Esg^+ and $Pdm1^+$), which continues to differentiate into an octoploid mature enterocyte (ECs; $Pdm1^+$).

B Quantification of luciferase activity after administration of endogenous chemicals. Error bars show the SD of six independent experiments.

C Immunofluorescence images of pH3 staining with the midgut section from the R4 region in 40-day flies and 40-day flies with ALA administration started at 26th day after fly eclosion. pH3 (red) staining was used to visualize the mitosis of ISCs.

D Immunofluorescence images of *esg*-GFP and Delta (Dl) staining with the midgut section from the R4 region in 40-day flies and 40-day flies with ALA administration after fly eclosion (lifelong administration). *esg*-GFP (green) indicates ISCs and their differentiating cells. Dl (red) staining was used to visualize ISCs.

E A cartoon illustrating the sorting of *esg*-GFP^+ cells using FACS for RT–qPCR analysis (see Materials and Methods Details).

Data information: DAPI-stained nuclei are shown in blue. Scale bars represent 25 μm (C) and 10 μm (D). Error bars represent SDs. Student's *t*-tests, *$P < 0.05$, **$P < 0.01$, and non-significant (NS) represents $P > 0.05$.
Source data are available online for this figure.

© 2020 The Authors

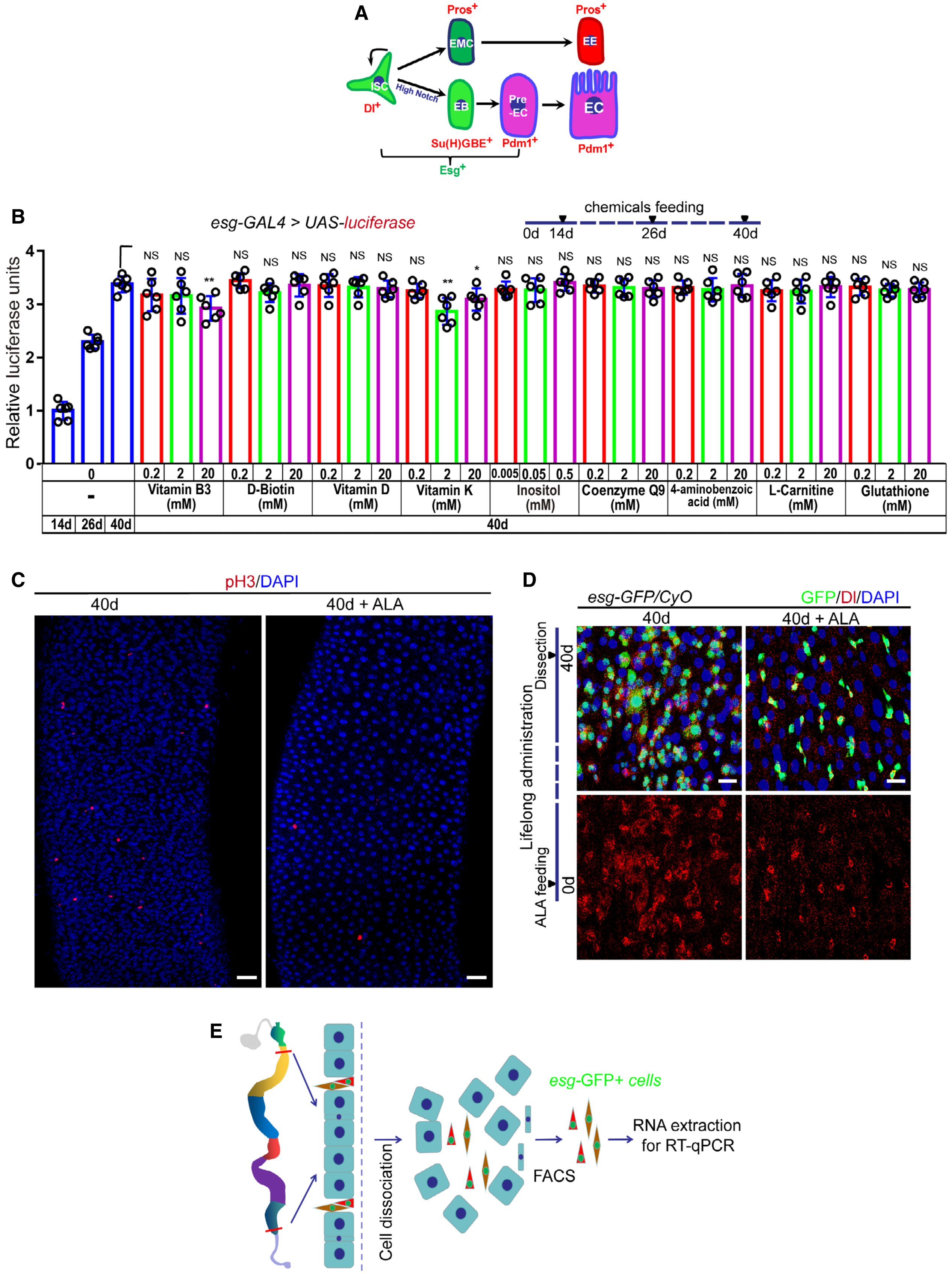

Figure EV1.

 © 2020 The Authors

Figure EV2. ALA synthesis reduces in aged flies and regulates lifespan of *Drosophila* (related to Figs 1–3).

A LC-ESI-MS/MS chromatogram of blank.
B LC-ESI-MS/MS chromatogram of ALA standard.
C Mass spectra of ALA.
D LC-ESI-MS/MS chromatogram of 8-aminooctanoic acid (internal standard).
E Mass spectra of 8-aminooctanoic acid (internal standard).
F *Las* transcript levels were reduced in *Las* RNAi (v22037) and *Las* RNAi (TH02737.N) flies, relative to their levels in control (*UAS-GFP*) flies. Error bars show the SD of three independent experiments.
G Food intake measured using the CAFE assay of *Drosophila* at 26th day with and without ALA administration as indicated. Error bars show the SD of three independent experiments.
H Survival (percentage) of female W^{1118} *Drosophila* with and without supplementation of ALA as indicated. The numbers of quantified flies: 200 (W^{1118} + 0 mM ALA) and 200 (W^{1118} + 0.5 mM ALA). The other two independent experiments related to Fig 3G.
I Survival (percentage) of female Canton-S *Drosophila* with and without supplementation of ALA as indicated. The numbers of quantified flies: 200 (Canton-S + 0 mM ALA) and 200 (Canton-S + 0.5 mM ALA). The other two independent experiments related to Fig 3I.
J Survival (percentage) of female *Canton-S Drosophila* with and without supplementation of ALA at 26-day as indicated. The numbers of quantified flies: 200 (*Canton-S* + 0 mM ALA) and 200 (*Canton-S* + 0.5 mM ALA). The other two independent experiments related to Fig 3J.

Data information: Error bars represent SDs. *P*-values for lifespan curves (H, I, and J) were calculated by the log-rank test. The statistical tests used in other panels were Student's *t*-tests. *$P < 0.05$, **$P < 0.01$, ***$P < 0.001$, ****$P < 0.0001$, and non-significant (NS) represents $P > 0.05$.
Source data are available online for this figure.

© 2020 The Authors

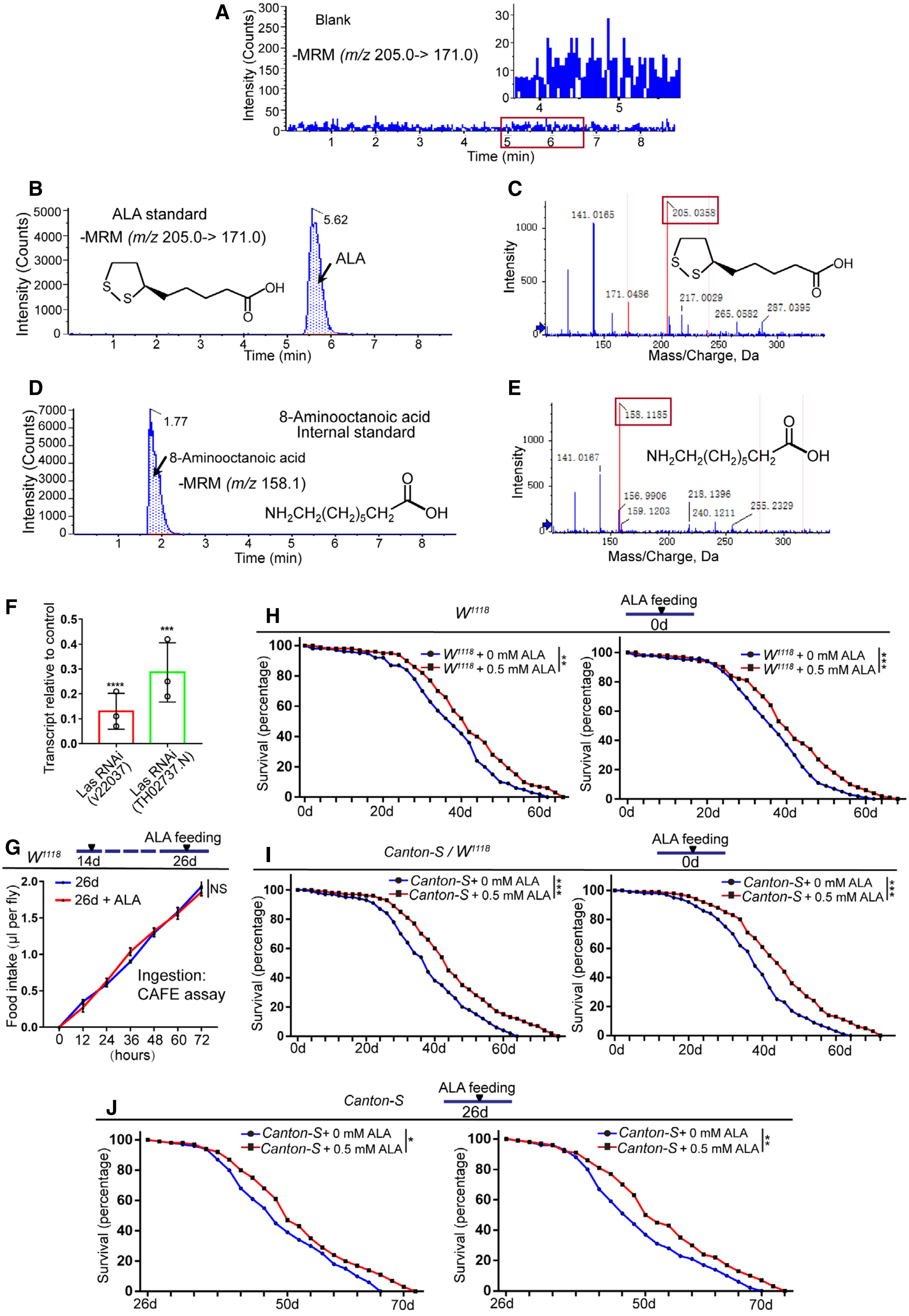

Figure EV2.

 © 2020 The Authors

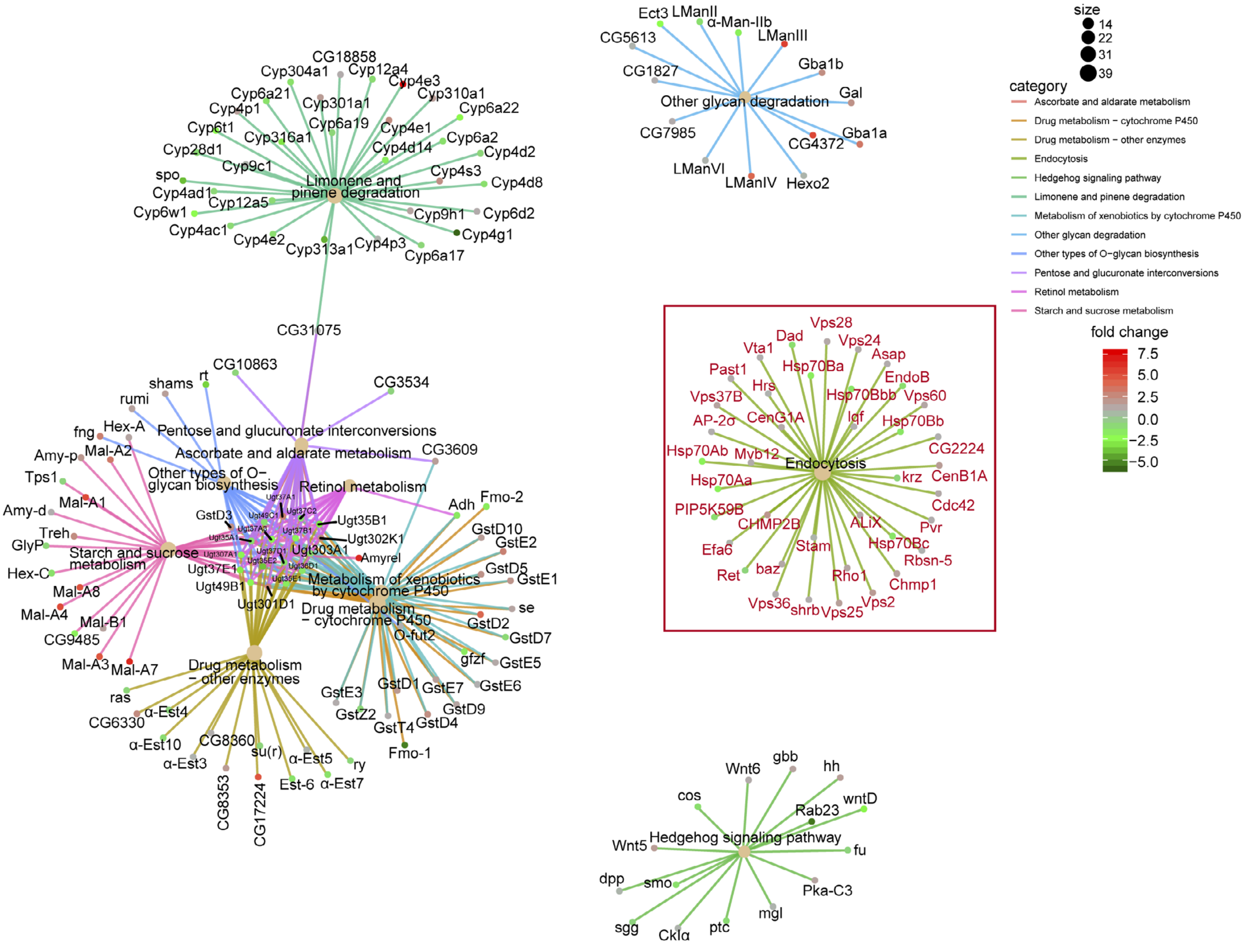

Figure EV3. KEGG pathway network analysis of RNA-seq (related to Fig 5).

KEGG pathway network analysis of RNA-seq in a pair-wise comparison of 40-day flies to 40-day flies treated with ALA. It showed significant enrichment of genes involved in the endocytosis process. The colored nodes represent pathways or target genes. Genes in the endocytosis pathway were marked red. The pathways were showed only by *P*-value < 0.05 and gene number > 10.

© 2020 The Authors

Figure EV4. ALA administration rejuvenates aged ISCs via activation of autophagy process (related to Figs 6 and 7).

A–E Expression of *esg-GAL4*-driven *UAS-GFP-mCherry-Atg8a* in 14-day *Drosophila* (A), 40-day *Drosophila* (B), 40-day *Drosophila* with ALA administration started at the middle age (26 days) (C), 14-day *Drosophila* carrying *esg^{ts}-GAL4*-driven *UAS-lacZ* (flies were cultured at 18°C and transferred to 29°C after flies eclosion) (D), and 14-day *Drosophila* with *Las* depleted in ISCs and EBs (flies were cultured at 18°C and transferred to 29°C after flies eclosion) (E). The boxed areas are enlarged at the right of the panel. GFP (green) and mCherry (white).

F, G Immunofluorescence images of midgut section from the R4 region in flies carrying *esg^{ts}-GAL4*-driven expression of *Atg8a* cDNA (F), *Atg8a* cDNA with ALA administration (G). Dl (red) staining was used to visualize ISCs.

H Quantification of the number of *esg*-GFP^{+} cells, Dl^{+} cells, pH3^{+} cells in *Drosophila* with indicated genotypes and manipulations. *n* is indicated. The numbers of quantified guts from left to right are 14, 17, 16, 16, 18, 14, 17, 16, 16, 18, 14, 17, 16, 16, and 18.

I Quantification of luciferase activity in *Drosophila* with indicated genotypes and manipulations. *n* is indicated. Error bars show the SD of six independent experiments.

J–L Immunofluorescence images of midgut section from the R4 region in *Drosophila* carrying *esg^{ts}-GAL4*-driven *lacZ* expression (J, control), *Atg5* expression (K), and *Atg5* expression with ALA administration (L). GFP (green) and Dl staining (red) was used to visualize ISCs.

M Survival (percentage) of female *Drosophila* with indicated genotypes. The numbers of quantified *Drosophila*: 200 (*UAS-lacZ*), 200 (*Las RNAi*), 200 (*Las RNAi + UAS-Atg5*), and 200 (*Las RNAi + UAS-Atg8a*). The other two independent experiments related to Fig 7N.

Data information: DAPI-stained nuclei are shown in blue. Scale bars represent 10 μm (A–G, and J–L). Error bars represent SDs. *P*-values for lifespan curves (M) were calculated by the log-rank test. The statistical tests used in other panels were Student's *t*-tests. *$P < 0.05$, **$P < 0.01$, ***$P < 0.001$, ****$P < 0.0001$, and non-significant (NS) represents $P > 0.05$.

Source data are available online for this figure.

 © 2020 The Authors

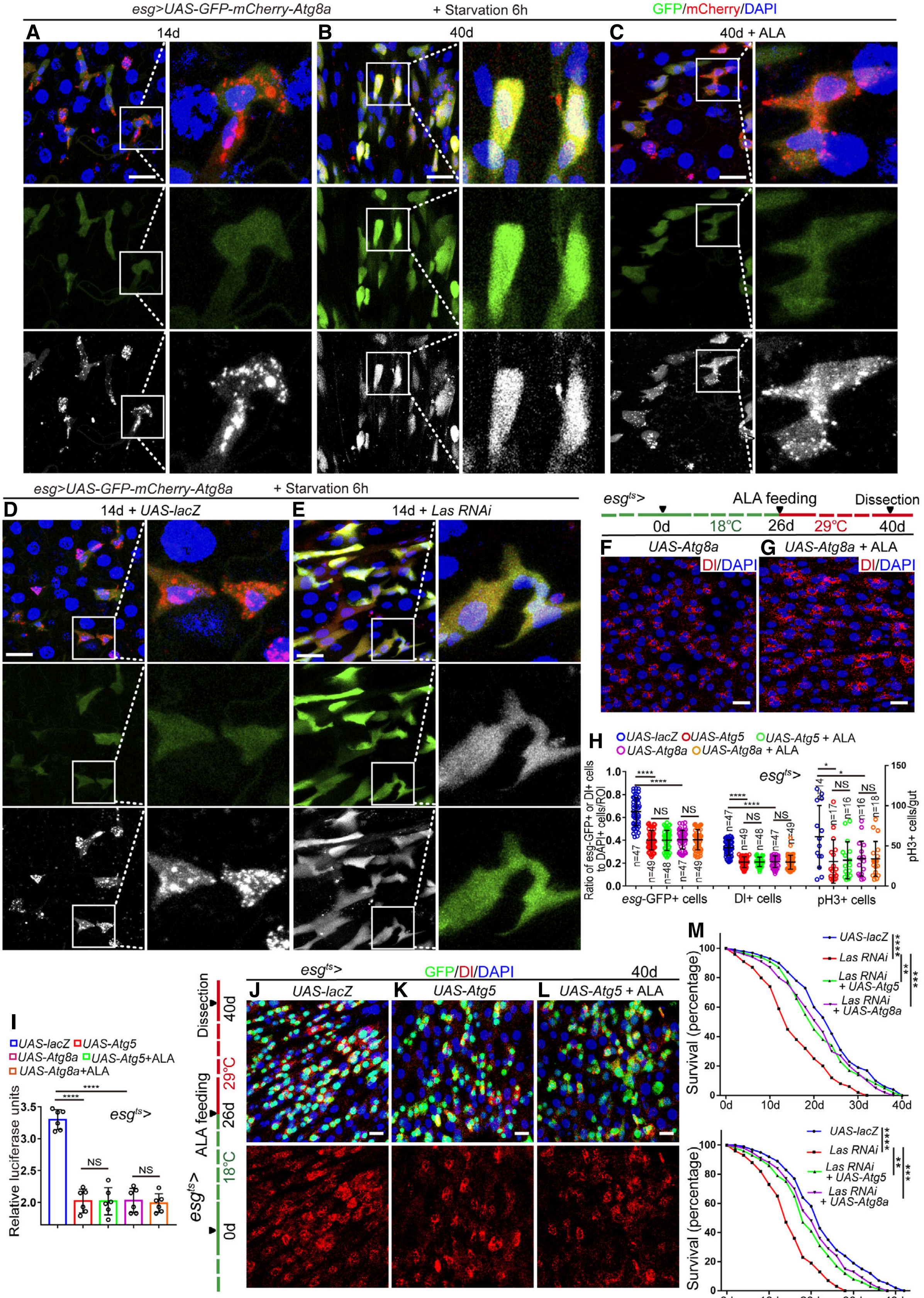
esg>UAS-GFP-mCherry-Atg8a
+ Starvation 6h
GFP/mCherry/DAPI
A
14d
B
40d
C
40d + ALA
esg>UAS-GFP-mCherry-Atg8a
+ Starvation 6h
D
14d + UAS-lacZ
E
14d + Las RNAi
esgts>
0d
18°C
ALA feeding
26d
29°C
Dissection
40d
F
UAS-Atg8a
G
UAS-Atg8a + ALA
Dl/DAPI
H
UAS-lacZ
UAS-Atg5
UAS-Atg5 + ALA
UAS-Atg8a
UAS-Atg8a + ALA
Ratio of esg-GFP+ or Dl+ cells to DAPI+ cells/ROI
pH3+ cells/gut
esg-GFP+ cells
Dl+ cells
pH3+ cells
NS
I
UAS-lacZ
UAS-Atg5
UAS-Atg8a
UAS-Atg5+ALA
UAS-Atg8a+ALA
Relative luciferase units
esgts>
NS
J
UAS-lacZ
K
UAS-Atg5
L
UAS-Atg5 + ALA
GFP/Dl/DAPI
M
UAS-lacZ
Las RNAi
Las RNAi + UAS-Atg5
Las RNAi + UAS-Atg8a
Survival (percentage)
0d
10d
20d
30d
40d

Figure EV4.

Figure EV5. ALA administration rejuvenates aged ISCs by modulating autophagy and endocytosis (related to Figs 6–10).

A–E Immunofluorescence images of midgut section from the R4 region in flies carrying *esg^{ts}-GAL4*-driven expression of *lacZ* cDNA (A), *Atg6 RNAi* (B), *Atg6 RNAi* with ALA administration (C), *Atg8a RNAi* (D), *Atg8a RNAi* with ALA administration (E). GFP (green), Dl (red) staining was used to visualize ISCs.

F Quantification of the number of *esg*-GFP^{+} cells, Dl^{+} cells, pH3^{+} cells in experiments (A–E). *n* is indicated. The numbers of quantified guts from left to right are: 16, 20, 19, 19, 20, 16, 20, 19, 19, 20, 16, 20, 19, 19, and 20.

G Quantification of luciferase activity of midguts in flies carrying *esg^{ts}-GAL4*-driven expression of *lacZ* cDNA, *Atg6 RNAi*, *Atg6 RNAi* with ALA administration, *Atg8a RNAi*, *Atg8a RNAi* with ALA administration. *n* is indicated. Error bars show the SD of six independent experiments.

H Quantification of three categories of midguts treated with the pH indicator Bromophenol blue in flies with indicated genotypes. The numbers of quantified guts from left to right are 90, 90, 90, 90, and 90. Error bars show the SD of three independent experiments.

I Quantification of excretion numbers from flies with indicated genotypes. Excretions are quantified in 30 fields for each group of 12 flies. Tests were repeated as three independent experiments.

J–N Immunofluorescence images of midgut section from the R4 region in flies carrying *esg^{ts}-GAL4*-driven expression of *lacZ* cDNA (J), *Rab5-DN* (K), *Rab5-DN* with ALA administration (L), *Rab7 RNAi* (M), *Rab7 RNAi* with ALA administration (N). GFP (green), Dl (red) staining was used to visualize ISCs.

O Survival (percentage) of female *Drosophila* with indicated genotypes. The numbers of quantified *Drosophila*: 200 (*UAS-lacZ*), 200 (*Las RNAi*), 200 (*Las RNAi + UAS-Rab5-CA*), and 200 (*Las RNAi + UAS-Rab7-CA*). The other two independent experiments related to Fig 10C.

P Quantification of three categories of midguts treated with the pH indicator Bromophenol blue in flies with indicated genotypes. The numbers of quantified guts from left to right are: 90, 90, 90, 90, and 90. Error bars show the SD of three independent experiments.

Q Quantification of excretion numbers from flies with indicated genotypes. Excretions are quantified in 30 fields for each group of 12 flies. Tests were repeated as three independent experiments.

Data information: DAPI-stained nuclei are shown in blue. Scale bars represent 10 μm (A–E, J–N). Error bars represent SDs. *P*-values for lifespan curves (O) were calculated by the log-rank test. The statistical tests used in other panels were Student's *t*-tests. $^{**}P < 0.01$, $^{***}P < 0.001$, $^{****}P < 0.0001$, and non-significant (NS) represents $P > 0.05$.
Source data are available online for this figure.

 © 2020 The Authors

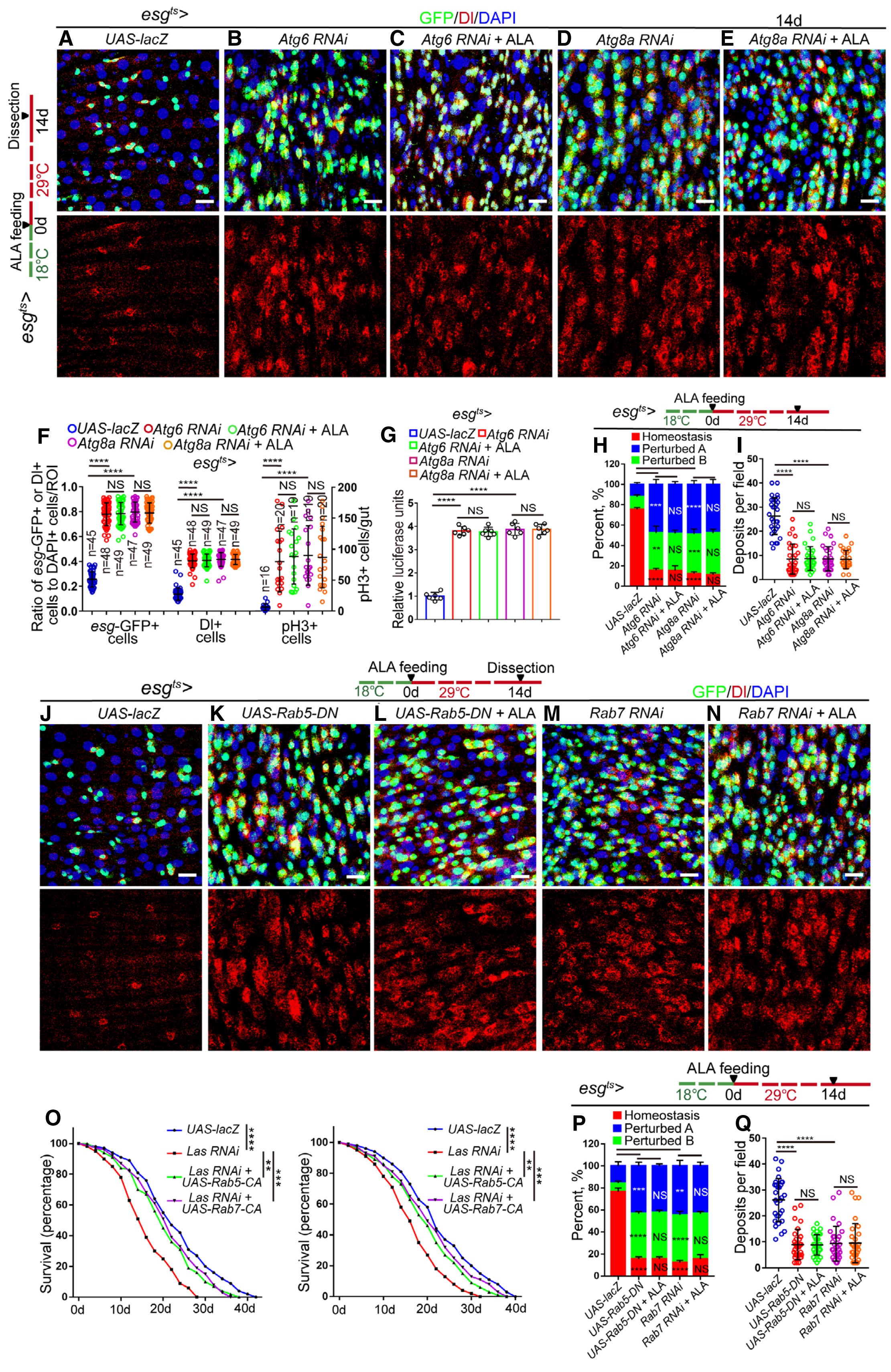

esg^ts>
GFP/Dl/DAPI
14d
A UAS-lacZ
B Atg6 RNAi
C Atg6 RNAi + ALA
D Atg8a RNAi
E Atg8a RNAi + ALA
Dissection
ALA feeding
18°C
29°C
0d
F
UAS-lacZ
Atg6 RNAi
Atg6 RNAi + ALA
Atg8a RNAi
Atg8a RNAi + ALA
Ratio of esg-GFP+ or Dl+ cells to DAPI+ cells/ROI
pH3+ cells/gut
esg-GFP+ cells
Dl+ cells
pH3+ cells
NS
G
Relative luciferase units
H
Homeostasis
Perturbed A
Perturbed B
Percent, %
I
Deposits per field
J UAS-lacZ
K UAS-Rab5-DN
L UAS-Rab5-DN + ALA
M Rab7 RNAi
N Rab7 RNAi + ALA
O
Survival (percentage)
Las RNAi
Las RNAi + UAS-Rab5-CA
Las RNAi + UAS-Rab7-CA
P
Q

Figure EV5.
